# Bellman–Steffensen type inequalities

**DOI:** 10.1186/s13660-018-1882-9

**Published:** 2018-10-22

**Authors:** Julije Jakšetić, Josip Pečarić, Ksenija Smoljak Kalamir

**Affiliations:** 1 Faculty of Mechanical Engineering and Naval Architecture, University of Zagreb, Zagreb, Croatia; 2 Faculty of Textile Technology, University of Zagreb, Zagreb, Croatia

**Keywords:** 26D15, 26D20, Steffensen’s inequality, Bellman–Steffensen type inequality, Measure theory, Exponential convexity

## Abstract

In this paper some Bellman–Steffensen type inequalities are generalized for positive measures. Using sublinearity of a class of convex functions and Jensen’s inequality, nonnormalized versions of Steffensen’s inequality are obtained. Further, linear functionals, from obtained Bellman–Steffensen type inequalities, are produced and their action on families of exponentially convex functions is studied.

## Introduction

Since its appearance in 1918 Steffensen’s inequality [1] has been a subject of investigation by many mathematicians because it plays an important role not only in the theory of inequalities but also in statistics, functional equations, time scales, special functions, etc. A comprehensive survey on generalizations and applications of Steffensen’s inequality can be found in [2].

In 1959 Bellman gave an $L^{p}$ generalization of Steffensen’s inequality (see [3]) for which Godunova, Levin and Čebaevskaya noted that it is incorrect as stated (see [4]). Further, in [5] Pečarić showed that the Bellman generalization of Steffensen’s inequality is true with very simple modifications of conditions. Using some substitutions in his result from [5], Pečarić also proved the following modification of Steffensen’s inequality in [6].

### Theorem 1.1

*Assume that two integrable functions* *f* *and* *G* *are defined on an interval* $[a,b]$, *f* *is nonincreasing*, *and*

1.1 $$ 0 \leq\lambda G(t) \leq \int_{a}^{b} G(t)\,dt, \quad\textit{for every } t\in[a,b], $$

*where* *λ* *is a positive number*. *Then*

1.2 $$ \frac{1}{\lambda} \int_{b-\lambda}^{b} f(t)\,dt \leq\frac{\int_{a}^{b} f(t)G(t)\,dt}{\int_{a}^{b} G(t)\,dt} \leq \frac{1}{\lambda} \int _{a}^{a+\lambda} f(t)\,dt. $$

In [7] Mitrinović and Pečarić gave necessary and sufficient conditions for inequality (1.2). The purpose of this paper is to generalize the aforementioned result for positive measures, using the approach from [8] and [9], and to give some applications related to exponential convexity.

Let $\mathcal{B}([a,b])$ be the Borel *σ*-algebra generated on $[a,b]$. In [10] the authors proved the following measure theoretic generalization of Steffensen’s inequality.

### Theorem 1.2

*Let* *μ* *be a positive finite measure on* $\mathcal{B}([a,b])$, *let* $g, h$ *and* *k* *be* *μ*-*integrable functions on* $[a,b]$ *such that* *k* *is positive and* *h* *is nonnegative*. *Let* *λ* *be a positive constant such that*

1.3 $$ \int_{[a,a+\lambda]} h(t)k(t)\,d\mu(t)= \int_{[a,b]} g(t)k(t)\,d\mu(t). $$

*The inequality*

1.4 $$ \int_{[a,b]} f(t)g(t)\,d\mu(t)\leq \int_{[a,a+\lambda]} f(t)h(t)\,d\mu(t) $$

*holds for every nonincreasing*, *right*-*continuous function* $f/k:[a,b]\rightarrow \mathbb{R}$ *if and only if*

1.5 $$ \int_{[a,x)} k(t)g(t)\,d\mu(t)\leq \int_{[a,x)} k(t)h(t)\,d\mu(t) \quad\textit{and}\quad \int_{[x,b]} k(t)g(t) \,d\mu(t)\geq0, $$

*for every* $x\in[a,b]$.

### Remark 1.1

In [10] the authors proved that if the function *f* is nonnegative, condition (1.3) can be replaced by the weaker condition

1.6 $$ \int_{[a,a+\lambda]} h(t) k(t) \,d\mu(t) \geq \int_{[a,b]} g(t) k(t) \,d\mu(t). $$

We note that if $f/k$ is nondecreasing, *f* and *h* are nonnegative, *k* is positive, and (1.6) holds, then the inequality in (1.4) is reversed. Furthermore, if $(f/k)(a)=0$, $f/k$ is increasing, *h* is nonnegative, *k* is positive, and (1.6) holds, then the inequality in (1.4) is reversed.

## Main results

Motivated by Theorem 1.1 and necessary and sufficient conditions given in [7], we prove some generalizations of Bellman–Steffensen type inequalities for positive measures.

### Theorem 2.1

*Let* *μ* *be a finite*, *positive measure on* $\mathcal{B}([a,b])$, *f*, *h* *be* *μ*-*integrable functions such that* *h* *is positive and* $f:[a,b]\rightarrow \mathbb{R}$ *nonincreasing*, *right*-*continuous*. *Then*

2.1 $$ \frac{\int_{[a,b]} f(t)G(t)\,d\mu(t)}{\int_{[a,b]} G(t)\,d\mu(t)}\leq \frac{\int_{[a,a+\lambda]} f(t)h(t)\,d\mu(t)}{\int_{[a,a+\lambda]} h(t)\,d\mu(t)} $$

*if and only if* $G:[a,b]\rightarrow \mathbb{R}$ *is* *μ*-*integrable and* *λ* *is a positive constant such that*

2.2 $$ \frac{\int_{[a,x)} G(t)\,d\mu(t)}{\int_{[a,b]} G(t)\,d\mu(t)}\leq \frac{\int_{[a,x)} h(t)\,d\mu(t)}{\int_{[a,a+\lambda]} h(t)\,d\mu (t)}\quad \textit{and}\quad \int_{[x,b]} G(t)\,d\mu(t)\geq0, $$

*for every* $x\in[a,b]$, *assuming* $\int_{[a,b]} G(t)\,d\mu(t)>0$.

*For a nondecreasing*, *right*-*continuous function* $f:[a,b]\rightarrow \mathbb{R}$, *inequality* (2.1) *is reversed*.

### Proof

*(Sufficiency)* Let us define the function

$$ g(t)=\frac{G(t)\int_{[a,a+\lambda]} h(t)\,d\mu(t)}{\int_{[a,b]} G(t)\,d\mu(t)}. $$

Since $\int_{[a,b]} g(t)\,d\mu(t)=\int_{[a,a+\lambda]} h(t)\,d\mu(t)$ and the conditions in (1.5) (for $k\equiv1$) are fulfilled, we can apply inequality (1.4) (for $k\equiv1$) and obtain inequality (2.1).

*(Necessity)* If we put the function

$$ f(t)= \textstyle\begin{cases} 1,& t< x;\\ 0,& t\geq x, \end{cases} $$

for $a\leq x\leq a+\lambda$ into inequality (2.1), we obtain the conditions in (2.2). □

In the following lemma we recall the property of sublinearity of the class of convex functions.

### Lemma 2.1

*If* $\phi:[0,\infty)\rightarrow \mathbb{R}$ *is a convex function such that* $\phi(0)=0$ *then for any* $0\leq a\leq1$

$$\phi(ax)\leq a\phi(x),\quad \textit{for any } x\in[0, \infty). $$

### Theorem 2.2

*Let* *μ* *be a finite*, *positive measure on* $\mathcal{B}([a,b])$. *Let* *f* *and* *h* *be nonnegative nonincreasing functions on* $[a,b]$, *and let* *ϕ* *be an increasing convex function on* $[0,\infty)$ *with* $\phi(0) = 0$. *If* *G* *is a nonnegative nondecreasing function on* $[a,b]$ *such that there exists a nonnegative function* $g_{1}$, *defined by the equation*

$$ \int_{[a,b]} g_{1}(t)\phi \biggl(\frac{G(t)}{g_{1}(t)} \biggr)\,d\mu (t)\leq \int_{[a,b]} h(t)\,d\mu(t) $$

*and* $\int_{[a,b]} g_{1}(t)\,d\mu(t)\leq1$, *then the following inequality is valid*:

$$ \phi \biggl(\frac{\int_{[a,b]} f(t)G(t)\,d\mu(t)}{\int_{[a,b]} G(t)\,d\mu(t)} \biggr)\leq \frac{\int_{[a,a+\lambda]} \phi(f(t))h(t)\,d\mu(t)}{\int _{[a,a+\lambda]} h(t)\,d\mu(t)}, $$

*where*

$$ \int_{[a,a+\lambda]} h(t)\,d\mu(t)=\phi \biggl( \int_{[a,b]} G(t)\,d\mu (t) \biggr). $$

### Proof

Using Jensen’s inequality, we have

$$ \phi \biggl(\frac{\int_{[a,b]} f(t)G(t)\,d\mu(t)}{\int_{[a,b]} G(t)\,d\mu(t)} \biggr)\leq\frac{\int_{[a,b]} \phi(f(t))G(t)\,d\mu (t)}{\int_{[a,b]} G(t)\,d\mu(t)}, $$

and since $\phi\circ f$ is nonincreasing, we only have to check conditions in (2.2). Since *G* is nonnegative, obviously $\int_{[x,b]} G(t)\,d\mu(t)\geq0$. So we only have to show

2.3 $$ \phi \biggl( \int_{[a,b]} G(t)\,d\mu(t) \biggr) \int_{[a,x)} G(t)\,d\mu (t)\leq \int_{[a,x)} h(t)\,d\mu(t) \int_{[a,b]} G(t)\,d\mu(t). $$

Using sublinearity from Lemma 2.1 and Jensen’s inequality, we have

2.4 $$\begin{aligned} \phi \biggl( \int_{[a,b]} G(t)\,d\mu(t) \biggr)&=\phi \biggl( \int _{[a,b]} g_{1}(t)\,d\mu(t)\frac{\int_{[a,b]} G(t)\,d\mu(t)}{\int_{[a,b]} g_{1}(t)\,d\mu(t)} \biggr) \\ &\leq \int_{[a,b]} g_{1}(t)\,d\mu(t)\phi \biggl( \frac{\int_{[a,b]} g_{1}(t)\frac{G(t)}{g_{1}(t)}\,d\mu(t)}{\int_{[a,b]} g_{1}(t)\,d\mu(t)} \biggr) \\ &\leq \int_{[a,b]} g_{1}(t)\phi \biggl(\frac{G(t)}{g_{1}(t)} \biggr)\,d\mu (t)\leq \int_{[a,b]} h(t) \,d\mu(t). \end{aligned}$$

Since *G* is a nonnegative nondecreasing function and *h* is a nonnegative nonincreasing function, we see that for each $x\in[a,b]$,

$$\frac{\int_{[a,x)} G(t)\,d\mu(t)}{\int_{[a,x)} h(t)\,d\mu(t)}\leq \frac{\int_{[a,b]} G(t)\,d\mu(t)}{\int_{[a,b]} h(t)\,d\mu(t)}, $$

i.e.,

$$\int_{[a,b]} h(t)\,d\mu(t) \int_{[a,x)} G(t)\,d\mu(t)\leq \int_{[a,x)} h(t)\,d\mu(t) \int_{[a,b]} G(t)\,d\mu(t), $$

so along with (2.4) we proved (2.3). Hence, the proof is completed. □

### Remark 2.1

In Theorems 2.1 and 2.2 we proved similar results to those obtained by Liu in [11] but we only need *μ* to be finite and positive instead of finite continuous and strictly increasing as in [11].

We continue with some more general Bellman–Steffensen type inequalities related to the function $f/k$.

### Theorem 2.3

*Let* *μ* *be a finite*, *positive measure on* $\mathcal{B}([a,b])$, $f, h$ *and* *k* *be* *μ*-*integrable functions such that* *h* *is nonnegative*, *k* *is positive and* $f/k:[a,b]\rightarrow \mathbb{R}$ *is nonincreasing*, *right*-*continuous*. *Then*

2.5 $$ \frac{\int_{[a,b]} f(t)G(t)\,d\mu(t)}{\int_{[a,b]} k(t)G(t)\,d\mu (t)}\leq \frac{\int_{[a,a+\lambda]} f(t)h(t)\,d\mu(t)}{\int_{[a,a+\lambda]} k(t)h(t)\,d\mu(t)} $$

*if and only if* $G:[a,b]\rightarrow \mathbb{R}$ *is a* *μ*-*integrable function and* *λ* *is a positive constant such that*

2.6 $$ \frac{\int_{[a,x)} k(t)G(t)\,d\mu(t)}{\int_{[a,b]} k(t)G(t)\,d\mu (t)}\leq \frac{\int_{[a,x)} k(t)h(t)\,d\mu(t)}{\int_{[a,a+\lambda]} k(t)h(t)\,d\mu(t)} \quad\textit{and}\quad \int_{[x,b]} k(t)G(t)\,d\mu(t)\geq0, $$

*for every* $x\in[a,b]$, *assuming* $\int_{[a,b]} k(t)G(t)\,d\mu(t)>0$.

*For a nondecreasing*, *right*-*continuous function* $f/k:[a,b]\rightarrow \mathbb{R}$ *inequality* (2.5) *is reversed*.

### Proof

*(Sufficiency)* Let us define the function

$$ g(t)=\frac{G(t)\int_{[a,a+\lambda]} k(t)h(t)\,d\mu(t)}{\int_{[a,b]} k(t)G(t)\,d\mu(t)}. $$

Since $\int_{[a,b]} k(t)g(t)\,d\mu(t)=\int_{[a,a+\lambda]} k(t)h(t)\,d\mu(t)$ and (1.5) are fulfilled, we can apply (1.4), and so (2.5) is valid.

*(Necessity)* If we put the function

$$ f(t)= \textstyle\begin{cases} k(t),& t< x;\\ 0,& t\geq x, \end{cases} $$

for $a\leq x\leq a+\lambda$ in (2.5), we get (2.6). □

### Theorem 2.4

*Let* *μ* *be a finite*, *positive measure on* $\mathcal{B}([a,b])$. *Let* *h* *and* $f/k$ *be nonnegative*, *nonincreasing functions on* $[a,b]$ *such that* *k* *is positive*, *and let* *ϕ* *be an increasing convex function on* $[0,\infty)$ *with* $\phi(0) = 0$. *If* *G* *is a nonnegative*, *nondecreasing function on* $[a,b]$ *such that there exists a nonnegative function* $g_{1}$ *defined by the equation*

$$ \int_{[a,b]} g_{1}(t)\phi \biggl(\frac{k(t)G(t)}{g_{1}(t)} \biggr)\,d\mu (t)\leq \int_{[a,b]} k(t)h(t)\,d\mu(t) $$

*and* $\int_{[a,b]} g_{1}(t)\,d\mu(t)\leq1$, *then the following inequality is valid*:

2.7 $$ \phi \biggl(\frac{\int_{[a,b]} f(t)G(t)\,d\mu(t)}{\int_{[a,b]} k(t)G(t)\,d\mu(t)} \biggr)\leq \frac{\int_{[a,a+\lambda]} \phi (\frac{f(t)}{k(t)} )k(t)h(t)\,d\mu(t)}{\int_{[a,a+\lambda]} k(t)h(t)\,d\mu(t)}, $$

*where*

2.8 $$ \int_{[a,a+\lambda]} k(t)h(t)\,d\mu(t)=\phi \biggl( \int_{[a,b]} k(t)G(t)\,d\mu(t) \biggr). $$

### Proof

Using Jensen’s inequality, we have

$$ \begin{aligned} \phi \biggl(\frac{\int_{[a,b]} f(t)G(t)\,d\mu(t)}{\int_{[a,b]} k(t)G(t)\,d\mu(t)} \biggr) &=\phi \biggl( \frac{\int_{[a,b]} \frac{f(t)}{k(t)}k(t)G(t)\,d\mu (t)}{\int_{[a,b]} k(t)G(t)\,d\mu(t)} \biggr) \\ &\leq\frac{\int_{[a,b]} \phi (\frac{f(t)}{k(t)} )k(t)G(t)\,d\mu(t)}{\int_{[a,b]} k(t)G(t)\,d\mu(t)}. \end{aligned} $$

From (2.5) for $f\mapsto(\phi\circ(f/k))\cdot k$, since $\phi\circ(f/k)$ is nonincreasing, we have

$$ \frac{\int_{[a,b]} \phi (\frac{f(t)}{k(t)} ) k(t)G(t)\,d\mu(t)}{\int_{[a,b]} k(t)G(t)\,d\mu(t)}\leq \frac{\int_{[a,a+\lambda]} \phi (\frac{f(t)}{k(t)} ) k(t)h(t)\,d\mu(t)}{\int_{[a,a+\lambda]} k(t)h(t)\,d\mu(t)} $$

if conditions in (2.6) are satisfied. Obviously, $\int_{[x,b]} k(t)G(t)\,d\mu(t)\geq0$ since *k* and *μ* are positive and *G* is nonnegative. Hence, we have to show

2.9 $$\begin{aligned} &\phi \biggl( \int_{[a,b]} k(t)G(t)\,d\mu(t) \biggr) \int_{[a,x)} k(t)G(t)\,d\mu(t) \\ &\quad \leq \int_{[a,b]} k(t)G(t)\,d\mu(t) \int_{[a,x)} k(t)h(t)\,d\mu(t). \end{aligned}$$

Using sublinearity from Lemma 2.1 and Jensen’s inequality, we have

2.10 $$\begin{aligned} \phi \biggl( \int_{[a,b]} k(t)G(t)\,d\mu(t) \biggr)&=\phi \biggl( \int _{[a,b]} g_{1}(t)\,d\mu(t) \frac{\int_{[a,b]} k(t)G(t)\,d\mu(t)}{\int_{[a,b]} g_{1}(t)\,d\mu (t)} \biggr) \\ &\leq \int_{[a,b]} g_{1}(t)\,d\mu(t)\phi \biggl( \frac{\int_{[a,b]} g_{1}(t)\frac{k(t)G(t)}{g_{1}(t)}\,d\mu(t)}{\int_{[a,b]} g_{1}(t)\,d\mu (t)} \biggr) \\ &\leq \int_{[a,b]} g_{1}(t)\phi \biggl(\frac{k(t)G(t)}{g_{1}(t)} \biggr)\,d\mu(t) \\ &\leq \int_{[a,b]} k(t)h(t) \,d\mu(t). \end{aligned}$$

Since *G* and *h* are nonnegative nondecreasing and *k* is positive, we have

$$\frac{\int_{[a,x)} k(t)G(t)\,d\mu(t)}{\int_{[a,x)} k(t)h(t)\,d\mu(t)} \leq\frac{\int_{[a,b]} k(t)G(t)\,d\mu(t)}{\int_{[a,b]} k(t)h(t) \,d\mu(t)}, $$

i.e.,

$$\int_{[a,b]} k(t)h(t) \,d\mu(t) \int_{[a,x)} k(t)G(t)\,d\mu(t) \leq \int_{[a,x)} k(t)h(t)\,d\mu(t) \int_{[a,b]} k(t)G(t)\,d\mu(t). $$

So, along with (2.10), we have proved (2.9). Hence the theorem is proved. □

## Applications

In this section we use classes of log-convex, exponentially convex and *n*-exponentially convex functions. Definitions and properties of these classes of functions can be found, e.g., in Pečarić, Proschan and Tong [12], Bernstein [13], Pečarić and Perić [14], and Jakšetić, Pečarić [15].

The following example will be useful in our applications.

### Example 3.1

(i) $f(x)=e^{\alpha x}$ is exponentially convex on $\mathbb{R}$, for any $\alpha\in \mathbb{R}$.
(ii) $g(x)=x^{-\alpha}$ is exponentially convex on $(0,\infty )$, for any $\alpha>0$.

The following families of functions given in the next two lemmas will be useful in constructing exponentially convex functions.

### Lemma 3.1

*Let* *k* *be a positive function and* $p\in \mathbb{R}$. *Let* $\varphi _{p}:(0,\infty)\rightarrow \mathbb{R}$ *be defined by*

3.1 $$ \varphi_{p}(x)= \textstyle\begin{cases} \frac{x^{p}}{p}k(x),& p\neq0;\\ k(x)\log x,& p=0. \end{cases} $$

*Then* $x\mapsto(\varphi_{p}/k)(x)$ *is increasing on* $(0,\infty)$ *for each* $p\in \mathbb{R}$ *and* $p\mapsto(\varphi_{p}/k)(x)$ *is exponentially convex on* $(0, \infty)$ *for each* $x\in(0, \infty)$.

### Proof

Since $\frac{d}{dx} (\frac{\varphi_{p}(x)}{k(x)} )=x^{p-1}>0$ on $(0,\infty)$ for each $p\in \mathbb{R}$ we have that $x\mapsto(\varphi_{p}/k)(x)$ is increasing on $(0,\infty)$. From Example 3.1 the mappings $p\mapsto e^{p\log x}$ and $p\mapsto\frac{1}{p}$ are exponentially convex, and since $p\mapsto\frac{x^{p}}{p}=e^{p\log x}\cdot\frac{1}{p}$, the second conclusion follows. □

Similarly we obtain the following lemma.

### Lemma 3.2

*For* $p\in \mathbb{R}$ *let* $\phi_{p}:[0,\infty)\rightarrow \mathbb{R}$ *be defined by*

3.2 $$ \phi_{p}(x)=\frac{x^{p}}{p(p-1)},\quad p>1. $$

*Then* $x\mapsto\phi_{p}(x)$ *is convex on* $[0,\infty)$ *for each* $p>1$ *and* $p\mapsto\phi_{p}(x)$ *and* $p\mapsto\phi_{p}'(x)$ *are exponentially convex on* $(1, \infty)$ *for each* $x\in[0,\infty)$.

Using the Bellman–Steffensen type inequality given by (2.5), under the assumptions of Theorem 2.3, we can define a linear functional $\mathfrak{L}$ by

3.3 $$ \mathfrak{L}(f)= \frac{\int_{[a,b]} f(t)G(t)\,d\mu(t)}{\int_{[a,b]} k(t)G(t)\,d\mu(t)} -\frac{\int_{[a,a+\lambda]} f(t)h(t)\,d\mu(t)}{\int_{[a,a+\lambda]} k(t)h(t)\,d\mu(t)}. $$

We have that the functional $\mathfrak{L}$ is nonnegative on the class of nondecreasing, right-continuous functions $f/k:[a,b]\rightarrow \mathbb{R}$.

### Theorem 3.1

*Let* $f\mapsto\mathfrak{L}(f)$ *be the linear functional defined by* (3.3) *and let* $\Phi: \mathbb{R}\rightarrow \mathbb{R}$ *be defined by*

$$ \Phi(p)=\mathfrak{L}(\varphi_{p}), $$

*where* $\varphi_{p}$ *is defined by* (3.1). *Then the following statements hold*:

(i) *The function* Φ *is continuous on* $\mathbb{R}$.
(ii) *If* $n\in \mathbb{N}$ *and* $p_{1},\ldots,p_{n}\in \mathbb{R}$ *are arbitrary*, *then the matrix*
  $$\biggl[\Phi \biggl(\frac{p_{j}+p_{k}}{2} \biggr) \biggr]_{j,k=1}^{n} $$
  *is positive semidefinite*.
(iii) *The function* Φ *is exponentially convex on* $\mathbb{R}$.
(iv) *The function* Φ *is log*-*convex on* $\mathbb{R}$.
(v) *If* $p,q,r\in \mathbb{R}$ *are such that* $p< q< r$, *then*
  $$ \Phi(q)^{r-p}\leq \Phi(p)^{r-q} \Phi(r)^{q-p}. $$

### Proof

(i) Continuity of the function $p\mapsto\Phi(p)$ is obvious for $p\in \mathbb{R}\setminus\{0\}$. For $p=0$ it is directly checked using Heine characterization.

(ii) Let $n\in \mathbb{N}$, $p_{i}\in \mathbb{R}$, $i = 1, \ldots, n$ be arbitrary. Let us define an auxiliary function $\Psi: (0,\infty) \rightarrow \mathbb{R}$ by

$$\Psi(x)=\sum_{j,k=1}^{n}\xi_{j} \xi_{k}\varphi_{\frac{p_{j}+p_{k}}{2}}(x). $$

Now

$$\biggl( \frac{\Psi(x)}{k(x)} \biggr)'= \sum _{j,k=1}^{n} \xi_{j} \xi_{k} x^{\frac{p_{j}+p_{k}}{2}-1}= \Biggl(\sum_{j=1}^{n} \xi_{j}x^{\frac{p_{j}-1}{2}} \Biggr)^{2}\geq0 $$

implies that $\Psi/k$ is nondecreasing on $(0,\infty)$, so $\mathfrak{L}(\Psi)\geq0$. This means that

$$\biggl[\Phi \biggl(\frac{p_{j}+p_{k}}{2} \biggr) \biggr]_{j,k=1}^{n} $$

is a positive semidefinite matrix.

Claims (iii), (iv), (v) are simple consequences of (i) and (ii). □

Let *k* be a positive function and let $\{\theta_{p}/k: p\in(0,\infty )\}$ be the family of functions defined on $[0,\infty)$ by

3.4 $$ \theta_{p}(x)=\frac{x^{p}}{p}k(x). $$

Similarly as in Lemma 3.1 we conclude that $x\mapsto (\theta_{p}/k)(x)$ is increasing on $[0,\infty)$ for each $p\in \mathbb{R}$ and $p\mapsto(\theta_{p}/k)(x)$ is exponentially convex on $(0, \infty)$ for each $x\in[0, \infty)$.

Let us define a linear functional $\mathfrak{M}$ by

3.5 $$ \mathfrak{M}(f)= \int_{[a,b]} f(t)g(t)\,d\mu(t) - \int_{[a,a+\lambda ]} f(t)h(t)\,d\mu(t). $$

Under the assumptions of Remark 1.1, we have that the linear functional $\mathfrak{M}$ is nonnegative acting on nondecreasing functions $f/k:[a,b]\rightarrow \mathbb{R}$ with the property $(f/k)(a)=0$.

### Theorem 3.2

*Let* $f\mapsto\mathfrak{M}(f)$ *be the linear functional defined by* (3.5) *and let* $F:(0,\infty)\rightarrow \mathbb{R}$ *be defined by*

$$ F(p)=\mathfrak{M}(\theta_{p}), $$

*where* $\theta_{p}$ *is defined by* (3.4). *Then the following statements hold*:

(i) *The function* *F* *is continuous on* $(0,\infty)$.
(ii) *If* $n\in \mathbb{N}$ *and* $p_{1},\ldots,p_{n}\in(0,\infty)$ *are arbitrary*, *then the matrix*
  $$\biggl[F \biggl(\frac{p_{j}+p_{k}}{2} \biggr) \biggr]_{j,k=1}^{n} $$
  *is positive semidefinite*.
(iii) *The function* *F* *is exponentially convex on* $(0,\infty)$.
(iv) *The function* *F* *is log*-*convex on* $(0,\infty)$.
(v) *If* $p,q,r\in(0,\infty)$ *are such that* $p< q< r$, *then*
  $$ F(q)^{r-p}\leq F(p)^{r-q} F(r)^{q-p}. $$

### Proof

(i) Continuity of the function $p\mapsto F(p)$ is obvious.

(ii) Let $n\in \mathbb{N}$, $p_{i}\in(0,\infty)$, $i = 1, \ldots, n$ be arbitrary. Let us define an auxiliary function $\Psi: [0,\infty) \rightarrow \mathbb{R}$ by

$$\Psi(x)=\sum_{j,k=1}^{n}\xi_{j} \xi_{k}\theta_{\frac{p_{j}+p_{k}}{2}}(x). $$

Now

$$\biggl(\frac{\Psi(x)}{k(x)} \biggr)'= \Biggl(\sum _{j=1}^{n}\xi _{j}x^{\frac{p_{j}-1}{2}} \Biggr)^{2}\geq0 $$

implies that $\Psi/k$ is nondecreasing on $[0,\infty)$ and nonnegative since $(\Psi/k)(0)=0$. Hence, $\mathfrak{M}(\Psi)\geq0$ and we conclude that

$$\biggl[F \biggl(\frac{p_{j}+p_{k}}{2} \biggr) \biggr]_{j,k=1}^{n} $$

is a positive semidefinite matrix.

Claims (iii), (iv), (v) are simple consequences of (i) and (ii). □

In the following theorem we give the Lagrange-type mean value theorem.

### Theorem 3.3

*Let* $f\mapsto\mathfrak{M}(f)$ *be the linear functional defined by* (3.5), *let* *k* *be a positive function on* $[a,b]$ *and* $\psi/k\in C^{1}[a,b]$ *such that* $(\psi/k)(a)=0$. *Then there exists* $\xi\in[a,b]$ *such that*

$$ \mathfrak{M}(\psi)= \biggl(\frac{\psi(\xi)}{k(\xi)} \biggr)' \mathfrak{M}(e_{1}), $$

*where* $e_{1}(x)=(x-a)k(x)$.

### Proof

Since $\psi/k\in C^{1}[a,b]$ there exist

$$m=\min_{x\in[a,b]} \frac{\psi'(x) k(x)-\psi(x)k'(x)}{k^{2}(x)} \quad\text{and}\quad M=\max _{x\in[a,b]}\frac{\psi'(x) k(x)-\psi(x)k'(x)}{k^{2}(x)}. $$

Denote

$$h_{1}(x)=M(x-a)k(x)-\psi(x) \quad\text{and}\quad h_{2}(x)= \psi(x)-m(x-a)k(x). $$

Then $(h_{1}/k)(a)=(h_{2}/k)(a)=0$ and

$$\begin{aligned} &\biggl(\frac{h_{1}(x)}{k(x)} \biggr)'=M-\frac{\psi'(x) k(x)-\psi (x)k'(x)}{k^{2}(x)}\geq0, \\ &\biggl(\frac{h_{2}(x)}{k(x)} \biggr)'=\frac{\psi'(x) k(x)-\psi (x)k'(x)}{k^{2}(x)}-m\geq0, \end{aligned}$$

so $h_{1}/k$ and $h_{2}/k$ are nondecreasing, nonnegative functions on $[a,b]$, which means that $\mathfrak{M}(h_{1}), \mathfrak{M}(h_{2})\geq0$, i.e.,

$$ m \mathfrak{M}(e_{1})\leq\mathfrak{M}(\psi)\leq M \mathfrak{M}(e_{1}). $$

If $\mathfrak{M}(e_{1})=0$, the proof is complete. If $\mathfrak {M}(e_{1})>0$, then

$$m\leq\frac{\mathfrak{M}(\psi)}{\mathfrak{M}(e_{1})}\leq M $$

and the existence of $\xi\in[a,b]$ follows. □

Using the standard Cauchy type mean value theorem, we obtain the following corollary.

### Corollary 3.1

*Let* $f\mapsto\mathfrak{M}(f)$ *be the linear functional defined by* (3.5), *let* *k* *be a positive function on* $[a,b]$ *and* $\psi_{1}/k, \psi_{2}/k\in C^{1}[a,b] $ *such that* $(\psi_{1}/k)(a)=(\psi _{2}/k)(a)=0$, *then there exists* $\xi\in[a,b]$ *such that*

3.6 $$ \frac{ (\frac{\psi_{1}(\xi)}{k(\xi)} )'}{ (\frac {\psi_{2}(\xi)}{k(\xi)} )'} =\frac{\mathfrak{M}(\psi_{1})}{\mathfrak{M}(\psi_{2})}, $$

*provided that the denominator on the right*-*hand side is nonzero*.

### Remark 3.1

By (3.6) we can define various means (assuming that the inverse of $(\psi_{1}/k)'/ (\psi_{2}/k)'$ exists). Hence,

3.7 $$ \xi= \biggl(\frac{ (\frac{\psi_{1}}{k} )'}{ (\frac {\psi_{2}}{k} )'} \biggr)^{-1} \biggl( \frac{\mathfrak{M}(\psi_{1})}{\mathfrak{M}(\psi_{2})} \biggr). $$

If we substitute $\psi_{1}(x) = \theta_{p}(x), \psi_{2}(x) = \theta _{q}(x)$ (where $\theta_{p}$ is defined by (3.4)) in (3.7) and use the continuous extension, we obtain the following expressions:

$$ M(p,q)= \textstyle\begin{cases} (\frac{\mathfrak{M}(\theta_{p})}{\mathfrak {M}(\theta_{q})} )^{\frac{1}{p-q}},& p\neq q; \\ \exp (\frac{\mathfrak{M}(\theta_{0}\theta_{p})}{\mathfrak {M}(\theta_{p})} -\frac{1}{p} ), &p=q, \end{cases} $$

where $\theta_{0}(x)=\log x$ and $p,q \in(0,\infty)$.

Using the characterization of convexity by the monotonicity of the first order divided differences, it follows (see [12, p. 4]): if $p, q, u, v \in(0,\infty)$ are such that $p\leq u, q\leq v$ then

$$ M(p,q)\leq M(u,v). $$

Using (2.7), under assumptions of Theorem 2.4, we can define a linear functional $\mathfrak{N}$ by

3.8 $$ \mathfrak{N}(\phi)= \frac{\int_{[a,a+\lambda]} \phi (\frac {f(t)}{k(t)} )k(t)h(t)\,d\mu(t)}{\int_{[a,a+\lambda]} k(t)h(t)\,d\mu(t)} -\phi \biggl( \frac{\int_{[a,b]} f(t)G(t)\,d\mu(t)}{\int_{[a,b]} k(t)G(t)\,d\mu(t)} \biggr). $$

We have that the linear functional $\mathfrak{N}$ is nonnegative on the class of increasing convex functions *ϕ* on $[0,\infty)$ with the property $\phi(0) = 0$.

### Theorem 3.4

*Let* $f\mapsto\mathfrak{N}(f)$ *be the linear functional defined by* (3.8) *and let* $H:(1,\infty)\rightarrow \mathbb{R}$ *be defined by*

$$ H(p)=\mathfrak{N}(\phi_{p}), $$

*where* $\phi_{p}$ *is defined by* (3.2). *Then the following statements hold*:

(i) *The function* *H* *is continuous on* $(1,\infty)$.
(ii) *If* $n\in \mathbb{N}$ *and* $p_{1},\ldots,p_{n}\in(1,\infty)$ *are arbitrary*, *then the matrix*
  $$\biggl[H \biggl(\frac{p_{j}+p_{k}}{2} \biggr) \biggr]_{j,k=1}^{n} $$
  *is positive semidefinite*.
(iii) *The function* *H* *is exponentially convex on* $(1,\infty)$.
(iv) *The function* *H* *is log*-*convex on* $(1,\infty)$.
(v) *If* $p,q,r\in(1,\infty)$ *are such that* $p< q< r$, *then*
  $$ H(q)^{r-p}\leq H(p)^{r-q} H(r)^{q-p}. $$

### Proof

(i) Continuity of the function $p\mapsto H(p)$ is obvious.

(ii) Let $n\in \mathbb{N}, p_{i}\in (1,\infty)\ (i = 1, \ldots, n)$ be arbitrary and define an auxiliary function $\psi: [0,\infty) \rightarrow \mathbb{R}$ by

3.9 $$ \psi(x)=\sum_{j,k=1}^{n} \xi_{j}\xi_{k}\phi_{\frac{p_{j}+p_{k}}{2}}(x). $$

Now

3.10 $$ \psi'(0)=\sum_{j,k=1}^{n} \xi_{j}\xi_{k}\phi'_{\frac{p_{j}+p_{k}}{2}}(0)=0. $$

Further,

3.11 $$ \psi''(x)= \Biggl(\sum _{j=1}^{n}\xi_{j}x^{\frac{p_{j}-2}{2}} \Biggr)^{2}\geq0. $$

Relations (3.10) and (3.11), together with $\psi (0)=0$, imply that *ψ* is a convex increasing function, and then

$$ \mathfrak{L}(\psi)\geq0. $$

This means that the matrix

$$\biggl[H \biggl(\frac{p_{j}+p_{k}}{2} \biggr) \biggr]_{j,k=1}^{n} $$

is positive semidefinite.

Claims (iii), (iv), (v) are simple consequences of (i) and (ii). □

Similar to Corollary 3.1 we also have the following corollary.

### Corollary 3.2

*Let* $f\mapsto\mathfrak{N}(f)$ *be the linear functional defined by* (3.8) *and* $\psi_{1}, \psi_{2}\in C^{2}[0,a] $ *such that* $\psi_{1}(0)=\psi_{2}(0)=\psi'_{1}(0)=\psi'_{2}(0)=0$ *and such that* $\psi _{2}''(x)$ *does not vanish for any value of* $x\in[0,a]$, *then there exists* $\xi\in[0,a]$ *such that*

3.12 $$ \frac{\psi_{1}''(\xi)}{\psi_{2}''(\xi)}=\frac{\mathfrak{N}(\psi _{1})}{\mathfrak{N}(\psi_{2})}, $$

*provided that the denominator on the right*-*hand side is nonzero*.

### Remark 3.2

By (3.12) we can define various means (assuming that the inverse of $\psi_{1}''/\psi_{2}''$ exists). That is,

3.13 $$ \xi= \biggl(\frac{\psi_{1}''}{\psi_{2}''} \biggr)^{-1} \biggl( \frac {\mathfrak{N}(\psi_{1})}{\mathfrak{N}(\psi_{2})} \biggr). $$

If we substitute $\psi_{1}(x) = \phi_{p}(x), \psi_{2}(x) = \phi_{q}(x)$ in (3.13) and use the continuous extension, we obtain the following expressions:

$$ N(p,q)= \textstyle\begin{cases} (\frac{\mathfrak{N}(\phi_{p})}{\mathfrak{N}(\phi _{q})} )^{\frac{1}{p-q}},&p\neq q; \\ \exp (\frac{\mathfrak{N}(\phi_{0}\phi_{p})}{\mathfrak{N}(\phi _{p})} + \frac{3-2p}{(p-1)(p-2)} ), &p=q, \end{cases} $$

where $\phi_{0}(x)=\log x$ and $p,q\in(1,\infty)$.

Again, by the monotonicity one has: if $p, q, u, v \in(1,\infty)$ are such that $p\leq u, q\leq v$ then

$$ N(p,q)\leq N(u,v). $$

For a fixed $n\geq2$, let us define

$$\mathcal{C}_{n}=\{\psi_{p}: p\in J \}, $$

a family of functions from $C([0,a])$ such that $\psi_{p}(0)=\psi '_{p}(0)=0$, and $p\mapsto\psi''_{p}(x)$ is *n*-exponentially convex in the Jensen sense on *J* for every $x\in [0,a]$.

### Theorem 3.5

*Let* $f\mapsto\mathfrak{N}(f)$ *be the linear functional defined by* (3.8) *and let* $S:J\rightarrow \mathbb{R}$ *be defined by*

$$ S(p)=\mathfrak{N}(\psi_{p}), $$

*where* $\psi_{p}\in\mathcal{C}_{n}$. *Then the following statements hold*:

(i) *S* *is n*-*exponentially convex in the Jensen sense on* *J*.
(ii) *If* *S* *is continuous on* *J*, *then it is* *n*-*exponentially convex on* *J* *and for* $p, q, r \in J$ *such that* $p < q < r$, *we have*
  $$ S(q)^{r-p}\leq S(p)^{r-q}S(r)^{q-p}. $$
(iii) *If* *S* *is positive and differentiable on* *J*, *then for every* $p, q, u,v \in J$ *such that* $p\leq u, q \leq v$, *we have*
  $$ \widetilde{M}(p,q)\leq\widetilde{M}(u,v), $$
  *where* $\widetilde{M}(p,q)$ *is defined by*
  $$ \widetilde{M}(p,q) = \textstyle\begin{cases} (\frac{S(p)}{S(q)} )^{\frac{1}{p-q}},& p\neq q; \\ \mathrm{exp} (\frac{\frac{d }{dp} (S(p) )}{S(p)} ), & p=q. \end{cases} $$

### Proof

(i) Choose any *n* points $\xi_{1},\ldots, \xi_{n}\in \mathbb{R}$, any $p_{1},\ldots, p_{n}\in J$. Define an auxiliary function $\Psi: [0,a] \rightarrow \mathbb{R}$ by

3.14 $$ \Psi(x)=\sum_{k,m=1}^{n} \xi_{k}\xi_{m}\psi_{\frac{p_{k}+p_{m}}{2}}(x). $$

Then $\Psi(0)=\Psi'(0)=0$ and

$$ \begin{aligned} \Psi''(x)=\sum _{k,m=1}^{n}\xi_{k}\xi_{m} \psi''_{\frac {p_{k}+p_{m}}{2}}(x)\geq0 \end{aligned} $$

by definition of $\mathcal{C}_{n}$. Hence, Ψ is an increasing convex function, and then $\mathfrak{L}(\Psi)\geq0$, which is equivalent to

$$\sum_{k,m=1}^{n}\xi_{k} \xi_{m} S \biggl({\frac{p_{k}+p_{m}}{2}} \biggr)\geq0. $$

(ii) Since *S* is continuous on *J*, then it is *n*-exponentially convex.

(iii) This is a consequence of the characterization of convexity by the monotonicity of the first order divided differences (see [12, p. 4]). □

We also can further refine previous results using divided differences. Let

$$\mathcal{D}=\{\chi_{p}: p\in J \}, $$

be a family of functions from $C([0,a])$ such that $\chi_{p}(0)=0, p\mapsto[x, y;\chi_{p}]$ is exponentially convex on *J* for every choice of two distinct points $x, y\in [0,a]$, and $p\mapsto [x_{0}, x_{1}, x_{2};\chi_{p}]$ is exponentially convex on *J* for every choice of three distinct points $x_{0}, x_{1}, x_{2}\in [0,a]$.

### Theorem 3.6

*Let* $f\mapsto\mathfrak{N}(f)$ *be the linear functional defined by* (3.8) *and let* $H:J\rightarrow \mathbb{R}$ *be defined by*

$$ H(p)=\mathfrak{N}(\chi_{p}), $$

*where* $\chi_{p}\in\mathcal{D}$. *Then the following statements hold*:

(i) *If* $n\in \mathbb{N}$ *and* $p_{1},\ldots,p_{n}\in \mathbb{R}$ *are arbitrary*, *then the matrix*
  $$\biggl[H \biggl(\frac{p_{k}+p_{m}}{2} \biggr) \biggr]_{k,m=1}^{n} $$
  *is positive semidefinite*.
(ii) *If the function* *H* *is continuous on* *J*, *then* *H* *is exponentially convex on* *J*.
(iii) *If* *H* *is positive and differentiable on* *J*, *then for every* $p, q, u,v \in J$ *such that* $p\leq u, q \leq v$, *we have*
  $$ \widehat{M}(p,q)\leq\widehat{M}(u,v), $$
  *where* $\widehat{M}(p,q)$ *is defined by*
  $$ \widehat{M}(p,q) = \textstyle\begin{cases} (\frac{H(p)}{H(q)} )^{\frac{1}{p-q}},& p\neq q; \\ \mathrm{exp} (\frac{\frac{d }{dp} (H(p) )}{H(p)} ), & p=q. \end{cases} $$

### Proof

(i) Let $n\in \mathbb{N}, p_{1},\ldots, p_{n}\in \mathbb{R}$ be arbitrary and define an auxiliary function $\Psi: [0,a] \rightarrow \mathbb{R}$ by

$$ \Psi(x)=\sum_{k,m=1}^{n} \xi_{k}\xi_{m}\chi_{\frac{p_{k}+p_{m}}{2}}(x). $$

Then

$$[x, y;\Psi]=\sum_{k,m=1}^{n} \xi_{k}\xi_{m}[x, y;\chi_{\frac {p_{k}+p_{m}}{2}}]\geq0 $$

by definition of $\mathcal{D}$ and exponential convexity. This implies that Ψ is a nondecreasing function on $[0,a]$. Similarly, $[x_{0}, x_{1}, x_{2};\Psi]\geq0$, for every choice of three distinct points $x_{0}, x_{1}, x_{2}\in [0,a]$. This implies that Ψ is a nondecreasing, convex function on $[0,a]$ such that $\Psi (0)=0$. Hence $\mathfrak{L}(\Psi)\geq0$, which is equivalent to

$$\sum_{k,m=1}^{n}\xi_{k} \xi_{m} H \biggl(\frac{p_{k}+p_{m}}{2} \biggr)\geq0. $$

(ii) This follows from part (i).

(iii) This is a consequence of the characterization of convexity by the monotonicity of the first order divided differences (see [12, p. 4]). □
